# A Telehealth-Delivered Tai Chi Intervention (TaiChi4Joint) for Managing Aromatase Inhibitor–Induced Arthralgia in Patients With Breast Cancer During COVID-19: Longitudinal Pilot Study

**DOI:** 10.2196/34995

**Published:** 2022-06-21

**Authors:** Sameh Gomaa, Carly West, Ana Maria Lopez, Tingting Zhan, Max Schnoll, Maysa Abu-Khalaf, Andrew Newberg, Kuang-Yi Wen

**Affiliations:** 1 Department of Medical Oncology Thomas Jefferson University Philadelphia, PA United States; 2 Department of Pharmacology & Experimental Therapeutics Thomas Jefferson University Philadelphia, PA United States; 3 Department of Integrative Medicine and Nutritional Sciences Thomas Jefferson University Philadelphia, PA United States

**Keywords:** breast cancer, arthralgia, tai chi, telehealth, pain, mind-body therapy

## Abstract

**Background:**

Estrogen receptor–positive breast cancer is the most common type of breast cancer in postmenopausal women. Aromatase inhibitors (AIs) are the endocrine therapy of choice recommended for these patients. Up to 50% of those treated with an AI develop arthralgia, often resulting in poor adherence and decreased quality of life.

**Objective:**

The study is a single-arm longitudinal pilot study aiming to evaluate the safety, feasibility, acceptability, and potential efficacy of *TaiChi4Joint*, a remotely delivered 12-week tai chi intervention designed to relieve AI-induced joint pain.

**Methods:**

Women diagnosed with stage 0-III breast cancer who received an AI for at least 2 months and reported arthralgia with a ≥4 score on a 0 to 10 scale for joint pain were eligible for study enrollment. Participants were encouraged to join tai chi classes delivered over Zoom three times a week for 12 weeks. Program engagement strategies included using a private Facebook study group and a Box cloud for archiving live class recordings. The program uses SMS text messaging and emails with periodic positive quotes and evidence-based information on tai chi for facilitating community bonding and class attendance. Participants were invited to complete the following assessments at baseline and at 1-, 2-, and 3-month intervals from study enrollment: Brief Pain Inventory, Western Ontario and McMaster University Osteoarthritis Index (WOMAC), The Australian Canadian Osteoarthritis Hand Index (AUSCAN), Fatigue Symptom Inventory, Hot Flash Related Daily Interference Scale (HFRDIS), Pittsburgh Sleep Quality Index (PSQI), and Center for Epidemiological Studies–Depression (CES-D).

**Results:**

A total of 55 eligible patients were invited to participate, and 39 (71%) consented and completed the baseline assessments. Participants attended 61% (median) of the suggested classes, with no tai chi–related adverse events reported. Of the 39 participants, 22 completed the 3-month follow-up assessment with a 56% retention rate. Study participants reported improvement from baseline compared to 3 months as follows (paired *t* test): Brief Pain Inventory (*P*<.001), AUSCAN pain subscale (*P*=.007), AUSCAN function subscale (*P*=.004), Fatigue Symptom Inventory (*P*=.004) and PSQI (*P*<.001), and HFRDIS (*P*=.02) and CES-D (*P*<.001). In particular, for our primary end point of interest, improvements in hip and knee symptoms, measured by WOMAC’s three subscales, were clinically meaningful and statistically significant when adjusted for multiple comparisons from baseline to 3 months post intervention.

**Conclusions:**

The COVID-19 global pandemic has resulted in the need to rethink how mind-body therapies can be delivered. This study demonstrated the feasibility, acceptability, and potential efficacy of a telehealth-based tai chi intervention for reducing AI-induced arthralgia. The intervention decreased patient-reported pain and stiffness, and improved sleep quality and depressive symptoms. Fully powered, large, telehealth-based tai chi trials for AI-associated arthralgia are needed considering our promising findings.

**Trial Registration:**

ClinicalTrials.gov NCT04716920; https://www.clinicaltrials.gov/ct2/show/NCT04716920

## Introduction

Breast cancer (BC) is the most common cancer among women in the United States, and hormone receptor–positive BC accounts for approximately two-thirds of all BC. For postmenopausal women with hormone receptor–positive BC [1], long-term use of an aromatase inhibitor (AI) for 5 to 10 years after primary surgical treatment has been demonstrated to prevent disease relapse and improve disease-free survival [2-4]. A meta-analysis revealed that the overall pooled prevalence rate of AI-induced arthralgia was 45.9% in postmenopausal hormone receptor–positive patients with BC [5]. AI-related arthralgia includes the new onset of worsening joint pain, stiffness, and impaired function. Arthralgia can affect any joints but primarily affects the hips, knees, and wrists/hands, with 70% of women describing their joint pain as moderate to severe [6]. Symptom onset is generally within 6 weeks to 12 months after AI initiation but can occur even longer, and symptoms may appear abruptly or increase gradually over time [6-8]. The clinical significance of AI-related arthralgia is that in addition to negatively affecting the quality of life [9] and daily activities [10], it can also decrease patients’ adherence to AI use. Arthralgia can lead up to 25% of patients with BC who stop AIs early and 50% of patients who have disruptions to their treatment regimen and schedule [6,10]. This data has important clinical implications, as nonadherence to AIs in early BC has been shown to negatively impact BC-related survival [10]. Other common symptoms during AI therapy include fatigue [11], hot flashes [12], insomnia [13], and depression [14,15], all of which can also adversely affect a patient’s quality of life and AI treatment adherence [15].

The approach to managing AI-related arthralgia is complex due to the absence of validated treatment standards for clinicians to use. They are complicated by limited knowledge regarding the physiological mechanisms involved. Unfortunately, pharmacological interventions to treat AI-related arthralgia have resulted in limited relief [16] and can produce undesirable side effects and physiologic dependence, especially in elderly patients. Lifestyle changes and complementary and alternative interventions, including supplementation, physical activities, and acupuncture, have been evaluated in a small number of studies with mixed results [16]. Thus, there is a growing interest in developing and assessing novel integrative programs to manage this adverse event and improve patient adherence to AI therapy.

Tai chi is a multidimensional mind-body therapy that integrates moderate physical activity, deep breathing, and meditation; additionally, it offers a promising approach to symptom management in cancer populations [17]. Tai chi has been practiced for centuries, and approximately 25% of the urban female population in the United States older than 50 years has practiced tai chi [18]. Tai chi forms consist of a series of upper- and lower-extremity movements performed in a particular choreographic manner. The interplay modes may provide a possible rationale for soft and connective tissues [17]. These movements may induce local biochemical changes that modulate blood circulation, improve muscle flexibility, intensify the action of the lymphatic system, and loosen adherent connective tissue. The loosening of the adherent connective tissue is thought to enhance the reuptake of local nociceptive and inflammatory mediators [17]. Randomized clinical trials (RCTs) have demonstrated the feasibility and efficacy of tai chi for women with BC. Studies have reported tai chi’s beneficial effects on psychological status (eg, depression or self-efficacy), physical function (eg, upper limb functional mobility), and other symptoms (eg, fatigue or sleep dysfunction) [19].

Furthermore, tai chi interventions designed specifically for individuals with pain-related conditions (eg, osteoarthritic knee pain) have significantly improved pain and physical functional outcomes [20]. However, there is lack of data regarding the use of tai chi interventions to target Al-induced arthralgia for patients with BC. Almost no tai chi interventions exist using a remote telehealth administration for any contexts. Telehealth or telemedicine uses telecommunications technologies for increased access to health care. Several advantages exist for telehealth, including the cost- and time-effectiveness, especially amid the current COVID-19 pandemic [21,22], as cancer survivors are at a higher risk of developing complications from COVID-19 [23,24]. Their risk of contracting COVID-19 must be reduced. New information and communication technology (eg, Zoom and WhatsApp) offers a convenient solution. Aligned with the social distancing and quarantine requirements, between December 2020 to July 2021, we conducted a pilot study that evaluated the feasibility, acceptability, and preliminary efficacy of a 12-week tai chi intervention (*TaiChi4Joint*). The study was aimed to reduce AI-induced arthralgia in women with BC, delivered remotely over the Zoom videoconferencing platform (Zoom Video Communications) facilitated by social media and SMS text messaging–enabled engagement strategies.

## Methods

### Study Eligibility and Study Procedures

Inclusion criteria for study participation included participants being ≥18 years of age, being able to speak/read English, having been diagnosed with stage 0-III BC, being postmenopausal (ie, no menses for at least 1 year), receiving an AI (anastrozole, letrozole, or exemestane) for at least 2 months, having joint pain that started or worsened after the initiation of AIs, reporting that their worst pain score in the prior week was ≥4 score on a 0 to 10 scale, and willing to adhere to all the study procedures.

Exclusion criteria included having another type of cancer that was diagnosed in the past 5 years; having uncontrolled cardiac, pulmonary, or infectious disease; having a BMI>40 kg/m^2^; currently attending any mind-body therapy classes (eg, tai chi or yoga); having joint pain due to an inflammatory arthritic condition; having surgery in the past 6 months; having a joint injection in the past 3 months; currently taking corticosteroids or opioids; or having discontinued or planning to discontinue their AI in the next 6 months.

A research team member screened potentially eligible patients via the electronic medical record or were referred by the patient’s treating medical oncologists. The study team connected with major support groups in the greater Philadelphia area to promote the study and potentially reach eligible participants. An informed consent form was signed, mailed, and returned by a study participant after reviewing the study rationale, intervention, and potential adverse effects with a research team member. The research team also provided remote assistance over the phone to ensure that the Zoom platform was successfully installed on the participant’s computer, tablet, or smartphone and that they could access classes on time. Participants were instructed to complete baseline, 1-month, 2-month, and 3-month surveys either by a mailed paper-based survey or via a REDCap online survey.

### Ethical Approval

This study with IRB control number 20G.093 was administratively approved by Thomas Jefferson University Institutional Review Board on January 30, 2020 by Board number 2405. Study accrual began in December 2020 and was completed in April 2021.

### TaiChi4Joint Study Intervention

Tai chi is a multicomponent practice that integrates physical, psychosocial, emotional, spiritual, and behavioral elements. Participants were invited to attend three tai chi classes per week for the 12-week study duration via the Zoom platform. Using a manualized approach, each class provided objectives and learning activities. The learning activities included sequentially learning a specific set of tai chi that included 24 movements with verification of skills attainment on a weekly basis. Each class began with relaxation exercises that had breathing exercises and qigong warm-ups. The instructor reviewed the previously learned techniques and introduced new movements following the relaxation session. The first 8 weeks of classes focused on mastery of single forms through multiple repetitions. The latter weeks emphasized consolidation of daily practice routines with natural breathing integrated into all classes. Scheduled classes ran from 45 to 60 minutes each and occurred 4 times per week, thus allowing patients to choose 3 classes that best fit their schedule. Participants were also encouraged to practice these tai chi classes through video recordings and spend 30 minutes daily on most days of the week self-practicing the technique they learned.

### Facebook and Text Messaging–Enabled Engagement Strategies

Participants were encouraged to join an optional Facebook private *TaiChi4joint* group that consists of instructional videos matching the progress of weekly classes for at-home practice and promotes peer support in tai chi engagement. We used the Facebook page and a Box shared cloud drive to share the instructional video and the recorded live tai chi sessions for participants who could not attend class or wanted additional practice. Weekly Facebook posts also contained evidence-based information about tai chi, the potential impact of tai chi in reducing pain, class reminders, and milestone celebrations (eg, birthday or program completion) to foster participant engagement and community building. Further, weekly text messages addressing class schedule, self-practice reminders, and brief positive quotes were proactively sent to participants to promote engagement and offer additional support.

### Outcome Measures

#### Patient Demographics and Treatment Information

We collected the following study subject demographics and BC-specific information: age, race, ethnicity, education, marital status, BC diagnosis and stage, treatment history, and AI medication type.

#### Feasibility

We aimed to retain 50% of eligible patients at the 3-month follow-up survey. The cutoff of 50% retention rate was chosen and derived from other similar pilot studies using mind-body therapies to mitigate arthralgia [24]. Feasibility was also measured by the participant’s class attendance, which the research team coordinator logged each session.

#### Acceptability

The acceptability of the study intervention was defined as a 60% consenting rate. Justification for a 60% consenting rate as acceptable was based on prior BC survivorship behavioral studies [25,26]. We also asked whether participants found the intervention helpful in reducing their pain.

#### Musculoskeletal Symptoms

As arthralgia primarily affects the hips and knees [6], we chose the Western Ontario and McMaster University Osteoarthritis Index (WOMAC) as the primary efficacy outcome. WOMAC measures lower-extremity joint symptoms (hips/knees) in the past 7 days in three domains: pain, stiffness, and physical function [27]. WOMAC has been recommended with great sensitivity for measuring changes in musculoskeletal symptoms in patients with BC receiving AIs [28].

#### Hand Pain and Physical Function

We used the Australian Canadian Osteoarthritis Hand Index (AUSCAN) to assess pain and physical function in the hands. AUSCAN has been reported to have excellent sensitivity and responsiveness in detecting and measuring AI-induced arthralgia [28].

#### Overall Pain

The Brief Pain Inventory (BPI) is a 14-item questionnaire developed for use in patients with cancer that assesses the worst pain, pain severity, and pain interference over the past week reported on a scale of 0 to 10. The BPI is the most common, valid, and reliable measure to assess pain in patients with cancer.

#### Fatigue

The Fatigue Symptom Inventory (FSI) measure is a 13-item self-report measure shown to be sensitive to assessing change in fatigue among patients with BC and with substantial internal consistency. An overall score was used.

#### Hot Flash

We used the Hot Flash Related Daily Interference Scale (HFRDIS) to measure the effect of hot flashes on the overall quality of life and nine specific activities: work, social activities, leisure activities, sleep, mood, concentration, relations with others, sexuality, and enjoyment of life [29].

#### Sleep Quality

We used the Pittsburgh Sleep Quality Index (PSQI) for a subjective sleep assessment, including multiple sleep-related variables over the preceding month [30].

#### Depressive Symptoms

We used the Center for Epidemiological Studies–Depression (CES-D) to assess depressive symptoms [31], and it has shown excellent internal consistency and validity in patients with cancer [32].

### Analysis

The statistical analysis was performed using R (R Foundation for Statistical Computing). Summary demographic statistics were calculated. Summary statistics of the end points, WOMAC subscales, AUSCAN subscales, BPI, FSI, HFRDIS, PSQI, and CES-D at baseline and at 1-month, 2-month, and 3-month follow-ups were calculated. Paired *t* tests of 3 months versus baseline were performed for each end point, and findings with 95% CIs are presented. For our primary end points of the WOMAC subscales, clinically meaningful changes in the subscales were evaluated using the criteria >1.5 out of 20 decrease for WOMAC pain, >0.6 out of 8 decrease for WOMAC stiffness, and >4.6 out of 68 decrease for WOMAC function score [32]. In addition, the linear time trends of end points were explored using latent class mixed-effect models with the R package lcmm. Up to 3 latent classes were considered, and the best number of latent classes was determined by the Bayesian information criterion.

## Results

### Patient Characteristics

The mean age of patients was 58 (SD 10.6) years. Of the 39 patients, 30 were Caucasian and 36 were at least college graduates. There were 31 patients with stage I or II, and had undergone mastectomy (n=22), radiation (n=27), or chemotherapy (n=23). Half of the participants (n=21) had been taking anastrozole. A total of 24 patients had been taking an AI for less than 3 years (Table 1).

**Table 1:** Participant characteristics.

Variable			Participants (N=39)
Age (years), mean (SD)			58.18 (10.60)
**Race, n (%)**			
	Caucasian	30 (77)	
	African American	7 (18)	
	Asian	1 (3)	
	Unreported	1 (3)	
**Ethnicity, n (%)**			
	Non-Hispanic	38 (97)	
	Hispanic	1 (3)	
**Marital status, n (%)**			
	Married	26 (67)	
	Single	7 (18)	
	Divorced	5 (13)	
	Widowed	1 (3)	
**Education level, n (%)**			
	High school	3 (8)	
	College graduate	16 (41)	
	Postgraduate school	20 (51)	
**Breast cancer stage, n (%)**			
	0	2 (5)	
	I	14 (36)	
	II	17 (44)	
	III	6 (15)	
**Surgery^a^, n (%)**			
	Mastectomy	22 (56)	
	Lumpectomy	15 (39)	
Radiation^a^, n (%)			27 (69)
Chemotherapy^a^, n (%)			23 (59)
**AI^b^ type, n (%)**			
	Letrozole	6 (15)	
	Anastrozole	21 (54)	
	Exemestane	9 (23)	
	Other	3 (8)	
**AI medication duration (years), n (%)**			
	≤1	12 (31)	
	1-3	12 (31)	
	3-6	12 (31)	
	Unreported	3 (8)	

^a^Treatment options do not add up to 39 due to individual treatment choices.

^b^AI: aromatase inhibitor.

### Acceptability

A total of 55 eligible patients were invited to participate, and 39 of the 55 (70.9%, 95% exact CI 57.1%-82.4%) patients consented to study participation and completed the baseline assessment, which exceeded the acceptability threshold of 60%. To seek participants’ feedback on the intervention, 14 of 22 participants who completed the final survey responded to our postintervention evaluation. All 14 perceived high satisfaction with the intervention, and 10 reported that the intervention was beneficial in relieving their pain.

### Feasibility

Of the 39 participants, 26 completed a 1-month follow-up, 21 completed a 2-month follow-up, and 22 completed the final 3-month follow-up, with a retention rate of 56% (95% exact CI 39.6%-72.2%) at the end of the study. Study attendance was calculated as the number of classes of the 36 suggested classes (12 weeks × 3 per week) each participant attended, ranging from 3% (2 classes) to 100% (36 classes), with a median value of 61% (22 classes). Of the 39 participants, 16 attended 18 (50%) or more classes during the 12-week study period.

### Change in Patient-Reported Outcomes During the Study Duration

Table 2 shows statistically significant improvement in all our study measures from baseline to the 3-month follow-up. Overall pain (BPI) was reduced from 5.04 to 2.69 (*P*<.001). Significant reductions also occurred in the pain subscale (from 6.64 to 3.52; *P*=.007) and function subscale (from 11.13 to 5.71; *P*=.004) of AUSCAN. Significant improvements were detected in fatigue (FSI from 53.40 to 29.74; *P*=.004), hot flashes (HFRDIS from 28.14 to 12.44; *P*=.02), sleep quality (PSQI from 18 to 11.1; *P*<.001), and depressive symptom (CES-D from 38.33 to 33.95; *P*=.03). In particular, the significant reductions in pain (from 8.05 to 5.05; *P*<.001), stiffness (from 4.36 to 2.33; *P*<.001), and function (from 23.28 to 11.67; *P*<.001) measured by WOMAC, detected using paired *t* tests, were clinically meaningful. The three *P* values for the WOMAC measurements were adjusted for multiple comparison using the Benjamini-Hochberg correction [33]. We also provide the linear time trends estimated using latent class mixed-effect models, with the three WOMAC measurements adjusted for multiple comparison, as referenced in Table 2.

**Table 2:** Change in patient-reported outcomes over time.

Measure (range)	Baseline (N=39), mean (SD)	1 month (n=25), mean (SD)	2 months (n=22), mean (SD)	3 months (n=21), mean (SD)	3-month change (95% CI)^a^	*P* value	Linear time trend detected by the latent class model	*P* value
WOMAC^b^ pain (0-20 maximal pain)	8.05 (4.19)	6.52 (3.70)	6.36 (4.82)	5.05 (3.89)	2.57 (1.27-3.87)	<.001^c^	–0.51	<.001^c^
WOMAC stiffness (0-8 maximal stiffness)	4.36 (1.74)	3.44 (1.61)	3.00 (1.75)	2.33 (1.65)	1.67 (0.81-2.52)	<.001^c^	–0.77	<.001^c^
WOMAC function (0-68 minimal function)	23.28 (14.21)	17.76 (12.36)	16.32 (13.81)	11.67 (12.70)	8.95 (5.65-12.25)	<.001^c^	–0.71	<.001^c^
BPI^d^ (0-10 worse pain)	5.04 (2.17)	4.22 (2.29)	4.16 (1.82)	2.69 (2.18)	2.25 (1.27-3.24)	<.001	–0.456	<.001
AUSCAN^e^ pain (0-50 worse pain)	6.64 (4.57)	6.00 (4.27)	4.77 (4.08)	3.52 (3.56)	2.33 (0.71-3.96)	.007	–0.642	<.001
AUSCAN function (0-90 minimal function)	11.13 (7.63)	8.80 (8.10)	7.45 (7.97)	5.71 (7.27)	4.48 (1.62-7.33)	.004	–0.794	<.001
FSI^f^ (0-130 worse fatigue)	53.40 (27.29)	40.04 (26.53)	36.94 (22.02)	29.74 (20.05)	17.53 (6.20-28.86)	.004	–0.61	<.001
HFRDIS^g^ (0-100 worse hot flash)	28.14 (25.46)	19.27 (22.89)	13.10 (14.25)	12.44 (13.78)	10.15 (2.04-18.26)	.02	For 28 patients: –0.176; for 11 patients: –1.235	For 28 patients: .14; for 11 patients: <.001
PSQI^h^ (0-21 lower sleep quality)	18.00 (5.86)	15.04 (6.52)	14.55 (5.70)	11.10 (6.40)	6.14 (3.32-8.96)	<.001	–0.626	<.001
CES-D^i^ (0-60 greater depressive symptom severity)	38.33 (5.61)	37.40 (4.53)	36.05 (4.46)	33.95 (4.97)	2.62 (0.26-4.97)	.03	–0.353	<.001

^a^Paired *t* test.

^b^WOMAC: Western Ontario and McMaster University Osteoarthritis Index.

^c^Adjusted for multiple comparison.

^d^BPI: Brief Pain Inventory.

^e^AUSCAN: Australian Canadian Osteoarthritis Hand Index.

^f^FSI: Fatigue Symptom Inventory.

^g^HFRDIS: Hot Flash Related Daily Interference Scale.

^h^PSQI: Pittsburgh Sleep Quality Index.

^i^CES-D: Center for Epidemiological Studies-Depression.

### Participant Feedback and Lessons Learned

Comments and suggestions from participants, the tai chi instructor, and our research team’s observation notes were qualitatively summarized and discussed to reach a consensus on each identified theme and areas for future intervention improvement.

#### Improved Pain and Stiffness

Participants reported that their pain or stiffness improved, with the change starting quickly after the first few weeks of the intervention. The reduction in pain also facilitated other improvements in daily activities.

Stiffness and pain decreased. I felt joints in knees and elbows move into my sockets at some point. I’m more limber and able to lift my knees higher to run faster and further.

Within 2 weeks, I had relief from the joint pain, especially my shoulder pain.

#### Better Relaxation and Balance

Breathing training and exercise were recognized as helpful in improving relaxation and calmness. Many participants also reported their balance was improved over time.

I particularly liked the breathing exercise at the beginning. I will continue to use it for relaxation and mindfulness.

My flexibility and balance have improved.

#### Value in Instructor Support and Facebook Group/Text Messaging Motivation

Our tai chi instructor was perceived as highly supportive and patient. A bonding relationship between the instructor and participants was observed. Archived class videos and motivational words and encouragements delivered in the Facebook private group and SMS text messaging were also reported as positive and helpful, motivating participant’s tai chi practice.

The instructor was amazing and showed extraordinary patience.

It was helpful to have all the classes on the FB page.

I enjoyed the occasional random words of kindness.

#### The Convenience of Virtual Classes

Participants commented on the ease and comfort of virtually attending our tai chi classes, overcoming in-person participation barriers such as travel burdens and demanding schedules.

The Zoom class made it easier to fit into my schedule.

I didn’t have to deal with parking.

#### The Limited Class Schedule

The main reason for not being able to attend scheduled classes was the participant’s conflicting schedules. Participants reported that their nonattendance generally reflected competing life demands rather than their lack of desire to attend classes. Due to the budget constraint of the pilot, the availability of our weekly offered courses was limited with a fixed schedule of four classes per week.

Would like to have more time options during the week for live classes.

Unfortunately, I have only been able to attend classes twice per week, and several times my schedule prevented me from one of them.

#### Challenges of Varying Skill Levels in the Same Class

For the pilot, we used rolling enrollment, so participants started the course as they enrolled. However, participants and the instructor observed and reported issues of participants with different levels of tai chi learning progression, reducing the efficiency of the class conduct.

There were beginners starting regularly, so the pace had to remain slow; it was difficult for me to move very slowly when I knew the next move.

#### The Initial Technological Difficulty, Bandwidth Limitation, and Online Streaming Challenges

We observed a learning curve for using the Zoom technology when participants initially started the intervention. Some of our older participants who joined our Zoom classes using their cellphones experienced viewing difficulties due to the small screen, their vision limitations, and interrupted internet signal. The problem of following the instructor’s side-to-side movements virtually was noted.

Having the class on Zoom presented some challenges initially.

It would have been helpful to have a third camera; it was difficult keeping perspectives –rights and lefts.

## Discussion

### A Telehealth-Delivered Tai Chi Intervention Is Feasible and Acceptable

AI-induced arthralgia can prohibit normal functioning and decrease affected patients’ quality of life. Clinical trial data show a 22% rate of AI discontinuation due to AI-induced arthralgia [12]. Real-world data suggest that the rate of AI-related arthralgia is as high as 50% [5], with minimal nonpharmacological options. Our findings support the feasibility, acceptability, and potential benefits of our *TaiChi4Joint* intervention and provide direction for future research. Due to COVID-19 social distancing constraints, our entire study implementation procedures were remotely conducted from the end of 2021 into early 2022. Concerning acceptability, 28 of the 39 eligible patients agreed to participate in our tai chi intervention delivered by Zoom. Many commented that the main reason they were attracted to joining was the convenience of modality, allowing them to attend classes from home during COVID-19. With regard to feasibility, 22 of the 39 participants who completed the baseline assessment completed the final assessment, and no safety issues were reported. The documented 61% average class attendance rate is not ideal, with less than 50% of participants attending half of the suggested class dosage. Many participants reported their nonattendance was generally due to competing work and family demands, conflicting with the schedule of the classes offered, as observed in the qualitative feedback. Offering a more flexible class schedule with different skill levels in future trials could also help to boost attendance rates by allowing participants to choose classes at a time more convenient for them.

### Tai Chi Is Effective in Relieving AI-Induced Symptoms

We also found statistically significant improvements in pain, stiffness, impaired function, fatigue, hot flash, sleep quality, and depressive symptoms. In particular, the change of the WOMAC subscale scores over time, our primary outcome of interest, was clinically meaningful based on prior literature. Qualitative findings, including participants’ perceived reduction in pain and stiffness, improvement in relaxation and balance, and the value of the instructor’s and the intervention’s support, could potentially have facilitated the quantitative change in outcomes observed in the study. Further, to counter potential digital divide challenges and improve participant engagement, the following strategies addressing our lessons learned might be helpful for future studies: include a user manual and training session practicing joining the videoconference, offer loaner iPads with cellular plans, use a multi-camera streaming setup, use closed session groups, and offer a more flexible class schedule. Our results demonstrate the potential of the *TaiChi4Joint* telehealth approach and support a fully powered RCT of *TaiChi4Joint* in the future.

### Limitations

This is a single-arm pilot study; this design was the most suitable to tackle the question of feasibility and acceptability. Study outcomes will need more scrutiny under an RCT design with a control group. This will be required to correct for possible selection bias or placebo effects. Selection bias might have been a cause for the study’s favorable results, as patients adhering to the investigation have obtained benefits derived from tai chi practice.

A longer intervention duration and a more extended period of follow-up post intervention might be needed to understand the long-term and maintenance effects of tai chi in reducing AI-induced arthralgia.

The majority of our sample were Caucasian and had a college degree, limiting the generalization of our findings. Innovative recruitment strategies will be needed in the future to ensure the sample is generalizable and to increase outreach and support to minorities.

### Conclusion

COVID-19 increased stress levels while reducing access to mind-body services in patients with cancer. Research on remote delivery of integrative, complementary, and alternative medicine health supports the feasibility and benefits of these services [22,34]. Our study used existing technologies to bring tai chi to patients’ homes. We found that tai chi reduced joint pain and stiffness; decreased fatigue; improved sleep quality, hot flash, and depressive symptoms; and improved functioning. Tai chi was well tolerated, and no adverse events specific to the study intervention occurred. Our lessons learned and strategies described could help inform the design of telehealth-based behavioral support. Future randomized controlled trials are needed to establish the comparative efficacy of the TaiChi4Joint intervention to improve outcomes related to AI-induced arthralgia.

## Abbreviations

AI: aromatase inhibitor
AUSCAN: Australian Canadian Osteoarthritis Hand Index
BC: breast cancer
BPI: Brief Pain Inventory
CES-D: Center for Epidemiological Studies–Depression
FSI: Fatigue Symptom Inventory
HFRDIS: Hot Flash Related Daily Interference Scale
PSQI: Pittsburgh Sleep Quality Index
RCT: randomized clinical trial
WOMAC: Western Ontario and McMaster University Osteoarthritis Index
