# Correction: A Telehealth-Delivered Tai Chi Intervention (TaiChi4Joint) for Managing Aromatase Inhibitor–Induced Arthralgia in Patients With Breast Cancer During COVID-19: Longitudinal Pilot Study

**DOI:** 10.2196/40830

**Published:** 2022-07-19

**Authors:** Sameh Gomaa, Carly West, Ana Maria Lopez, Tingting Zhan, Max Schnoll, Maysa Abu-Khalaf, Andrew Newberg, Kuang-Yi Wen

**Affiliations:** 1 Department of Medical Oncology Thomas Jefferson University Philadelphia, PA United States; 2 Department of Pharmacology & Experimental Therapeutics Thomas Jefferson University Philadelphia, PA United States; 3 Department of Integrative Medicine and Nutritional Sciences Thomas Jefferson University Philadelphia, PA United States

In “A Telehealth-Delivered Tai Chi Intervention (TaiChi4Joint) for Managing Aromatase Inhibitor–Induced Arthralgia in Patients With Breast Cancer During COVID-19: Longitudinal Pilot Study” (JMIR Form Res 2022;6(6): e34995), the authors made the following corrections in the corresponding authorship:

1. In the originally published article, author Sameh Gomaa was erroneously listed as the corresponding author. The corresponding authorship is now correctly attributed to Kuang-Yi Wen.

2. Accordingly, the address and contact details of the corresponding author have been changed as follows:

Kuang-Yi Wen, PhD

Department of Medical Oncology

Thomas Jefferson University

834 Chestnut Street Suite 300

Philadelphia, PA, 19106

United States

Phone: 1 2155034623

3. In the originally published article, author Tingting Zhan was incorrectly associated with the following affiliation:

Department of Medical Oncology, Thomas Jefferson University, Philadelphia, PA, United States

The author is now correctly associated with newly added Affiliation 2:

Department of Pharmacology & Experimental Therapeutics, Thomas Jefferson University, Philadelphia, PA, United States

4. In the originally published article, author Andrew Newberg was incorrectly associated with the following affiliation:

Department of Medical Oncology, Thomas Jefferson University, Philadelphia, PA, United States

The author is now correctly associated with newly added Affiliation 3:

Department of Integrative Medicine and Nutritional Sciences, Thomas Jefferson University, Philadelphia, PA, United States

The correction will appear in the online version of the paper on the JMIR Publications website on July 19, 2022, together with the publication of this correction notice. Because this was made after submission to PubMed, PubMed Central, and other full-text repositories, the corrected article has also been resubmitted to those repositories.

